# Validity of the Medical College Admission Test for predicting MD–PhD student outcomes

**DOI:** 10.1007/s10459-015-9609-x

**Published:** 2015-05-08

**Authors:** James L. Bills, Jacob VanHouten, Michelle M. Grundy, Roger Chalkley, Terence S. Dermody

**Affiliations:** Medical Scientist Training Program, Vanderbilt University School of Medicine, D7235 Medical Center North, 1161 21st Avenue South, Nashville, TN 37232-2581 USA; Department of Biomedical Informatics, Vanderbilt University School of Medicine, Nashville, TN 37232 USA; Department of Biostatistics, Vanderbilt University School of Medicine, Nashville, TN 37232 USA; Department of Medical Education and Administration, Vanderbilt University School of Medicine, Nashville, TN 37232 USA; Department of Pathology, Microbiology, and Immunology, Vanderbilt University School of Medicine, Nashville, TN 37232 USA; Department of Pediatrics, Vanderbilt University School of Medicine, Nashville, TN 37232 USA; Elizabeth B. Lamb Center for Pediatric Research, Vanderbilt University School of Medicine, Nashville, TN 37232 USA

**Keywords:** MCAT, MD–PhD training, Predictors of outcomes, MCAT as predictor

## Abstract

The Medical College Admission Test (MCAT) is a quantitative metric used by MD and MD–PhD programs to evaluate applicants for admission. This study assessed the validity of the MCAT in predicting training performance measures and career outcomes for MD–PhD students at a single institution. The study population consisted of 153 graduates of the Vanderbilt Medical Scientist Training Program (combined MD–PhD program) who matriculated between 1963 and 2003 and completed dual-degree training. This population was divided into three cohorts corresponding to the version of the MCAT taken at the time of application. Multivariable regression (logistic for binary outcomes and linear for continuous outcomes) was used to analyze factors associated with outcome measures. The MCAT score and undergraduate GPA (uGPA) were treated as independent variables; medical and graduate school grades, time-to-PhD defense, USMLE scores, publication number, and career outcome were dependent variables. For cohort 1 (1963–1977), MCAT score was not associated with any assessed outcome, although uGPA was associated with medical school preclinical GPA and graduate school GPA (gsGPA). For cohort 2 (1978–1991), MCAT score was associated with USMLE Step II score and inversely correlated with publication number, and uGPA was associated with preclinical GPA (mspGPA) and clinical GPA (mscGPA). For cohort 3 (1992–2003), the MCAT score was associated with mscGPA, and uGPA was associated with gsGPA. Overall, MCAT score and uGPA were inconsistent or weak predictors of training metrics and career outcomes for this population of MD–PhD students.

## Introduction

Training physician-scientists is an important educational goal (Whitcomb 2007; Miller 2009; Wiley 2010). In 2014, 134 accredited medical schools in the United States and Canada offered some form of MD–PhD training (Association of American Medical Colleges n.d.a.). The application process for those seeking dual-degree training is rigorous and competitive, with the objective being to identify individuals with the potential to become physician-scientists who will establish careers in biomedical research (Andriole et al. 2008). Within the MD–PhD training population, students graduating from National Institutes of Health-supported Medical Scientist Training Programs (MSTPs) are among the most competitive and productive (Ley and Rosenberg 2005; Ahn et al. 2007; Brass et al. 2010). The first three MSTPs were funded by the NIH in 1964 (National Research Council 2005) with the goal of providing scholarships to those Leon Rosenberg referred to as “unusually committed” medical students (Rosenberg 2008). The rationale for these programs is that MD–PhD graduates should bring unique “insights of…clinical experience to…research and vice versa” (Santoro et al. 2007). As of 2014, the NIH provides funding for 44 MSTPs (National Institute of General Medical Sciences 2014).

MD–PhD program applicants are evaluated by most programs using the AMCAS application that contains a combination of quantitative data, including undergraduate grade point average (uGPA) and Medical College Admission Test (MCAT) scores, and qualitative data, consisting of personal essays, documentation of research and clinical experiences, description of leadership experiences, and letters of recommendation. However, specific components of applicant review and admissions criteria vary from program to program (Association of American Medical Colleges 2014; Association of American Medical Colleges n.d.a.).

The MCAT is an important evaluative tool for MD–PhD program admissions committees. The first standardized examination for medical school entrance was developed in 1928 in response to high medical school attrition rates as a means to assess more objectively an applicant’s aptitude for a career in medicine (Collier 2011). The MCAT has undergone four subsequent revisions—in 1946, 1962, 1977, and 1991—with the next version due for implementation in 2015 (McGaghie 2002; Dienstag 2011).

The MCAT has been the focus of a number of studies to assess whether the test predicts medical school performance (Gough and Hall 1975; Mitchell 1990, Swanson et al. 1996; Wiley and Koenig 1996; Koenig et al. 1998; Hojat et al. 2000; Gilbert et al. 2002; Evans and Wen 2007; Kreiter and Kreiter 2007; Kuncel and Hezlett 2007; Callahan et al. 2010; Dunleavy et al. 2013). As Julian (2005) summarized in her study, medical school “grades were best predicted by a combination of MCAT scores and uGPAs, with MCAT scores providing a substantial increment over uGPAs. MCAT scores were better predictors of USMLE scores than were uGPAs, and the combination did little better than MCAT scores alone.” However, Donnon et al. (2007) reported in a meta-analysis of same-subject studies, including the study by Julian, that “the predictive validity of the MCAT ranges from small to medium for both medical school performance and medical board licensing exam measures.” Other studies have examined the predictive value of the MCAT when evaluating outcomes with subjective components such as clinical grades, in which little correlation was observed (Roberts et al. 2012), and unimpeded progress through undergraduate medical training, in which MCAT score combined with uGPA yielded the strongest correlation (Dunleavy et al. 2013).

To our knowledge, there are no reports of the predictive value of the MCAT for the training performance of MD–PhD students. In addition to the common metrics used to evaluate applicants for medical school, MD–PhD applicants are evaluated more rigorously for research aptitude and experiences, which may not be accurately assessed by the MCAT. Nonetheless, the MCAT score may be used by MD–PhD programs to differentiate applicants, especially if several attributes are comparable. Average MCAT scores of various student populations are also used by funding agencies and external evaluation groups as a metric to judge the quality of a program and the caliber of matriculating students. The goal of our study was to determine whether the MCAT predicts training performance measures and career outcomes among MD–PhD students from a single institution.

## Methods

### Purpose

This study was designed to determine the predictive power of the MCAT for a variety of MD–PhD student training outcomes. Outcomes unique to MD–PhD students, and the focus of this study, are grades during graduate training, publications resulting from graduate research, and the career outcome as determined by the first position following completion of residency or fellowship. For a subset of students, we also examined time-to-PhD defense and success in procuring an independent predoctoral fellowship. Outcomes common with MD students are grades in the preclinical and clinical years of medical training and performance on individual steps of the USMLE. The null hypothesis was that MCAT scores of MD–PhD students do not correlate with these outcome measures.

### Design and setting

A database was constructed for this study that included all Vanderbilt MSTP graduates who had matriculated from 1963 through 2003 (N = 153) for whom we had collected MCAT, uGPA, medical school GPA (msGPA), graduate school GPA (gsGPA), USMLE scores, length of time to PhD defense, receipt of a predoctoral fellowship, research publications, and type of first career position. Data sources were the Vanderbilt University School of Medicine (VUSM) Center for Outcomes Research in Education, VUSM Registrar’s Office, VUSM Medical School Archives, and VUSM Biomedical Research, Education, and Training Office. Other sources were the Association of American Medical Colleges Data Committee and National Board of Medical Examiners. We searched for publications via the US National Library of Medicine on-line journal repository at www.PubMed.gov. Time-to-PhD defense was obtained from a database maintained by the VUSM Office for Biomedical Research, Education, and Training. First post-residency position of employment was obtained from the Vanderbilt MSTP alumni database.

Data were stored at all times on a secure server in a password-protected file accessible only to the principal investigators. The institutional review board at VUSM (Nashville, Tennessee) approved this study with a waiver of consent given the stipulation of data protection.

The first cohort of MSTP students matriculated from 1963 to 1977 and had taken the pre-1978 version of the MCAT (N = 32). The second cohort of students matriculated from 1978 to 1991 and had taken the 1977 version of the MCAT (N = 65). The third cohort of students matriculated from 1992 to 2003 and had taken the current version of the MCAT (N = 56).

Approximately 90 % of students who enrolled in the MSTP at Vanderbilt completed the first 2 years of medical school, 3–5 years of graduate school to earn a PhD degree, and the last 2 years of medical school to complete the MD degree. The remaining students either transitioned into PhD research training after the first year of medical school or completed 3 years of medical school prior to initiating PhD studies. Neither of the latter options was routinely exercised.

### Predictor variables

The MCAT for students who matriculated from 1963 to 1977 consisted of subcategories of Science Achievement, General Information, Quantitative Ability, and Verbal Ability (McGaghie 2002; Callahan et al. 2010). Total scores were reported in a range between 200 and 800. The MCAT for students who matriculated from 1978 to 1991 consisted of subcategories of Biology, Chemistry, Physics, Science Problem Solving, Quantitative Skills, and Reading Skills. We analyzed only the composite overall score for each applicant. The MCAT examination for students who matriculated from 1992 to 2003 consisted of subcategories of Biological Sciences, Physical Sciences, and Verbal Reasoning. The Biological and Physical Sciences sub-tests were scored from 1 to 15 and Verbal Reasoning from 1 to 13 (changed to 1–15 in 2003) (McGaghie 2002). We used the aggregate score of the three primary subtests for initial analysis and performed additional analyses of the third cohort using the Biological Sciences, Physical Sciences, and Verbal Reasoning subscores. We did not include in our analyses the writing component of this version of the MCAT, as previous studies suggest that it has limited predictive validity (Gilbert et al. 2002; Kreiter and Kreiter 2007; Flanigan and Morse 2014). We did not separate science and non-science categories of the uGPA. Descriptive statistics for the independent variables (MCAT score and uGPA) and dependent variables used as outcome measures are provided in Table 1. We also determined whether the combination of MCAT score and uGPA resulted in increased predictive validity.

**Table 1:** Descriptive statistics for the data set of 153 graduates of the Vanderbilt Medical Scientist Training Program who matriculated from 1963 to 2003

Cohort (Matriculation years) (N)	MCAT	uGPA	gsGPA	Fellowship	Pubs	Time-to-PhD defense	Career outcome	mspGPA	mscGPA	Step I	Step II	Step III
Cohort 1 (1963–1977) (N = 32)												
Mean	560	3.46	3.55	–	6.5	–	0.79	3.45	3.54	211.1	215.0	220.6
SD	83.6	0.42	0.33	–	4.7	–	0.42	0.19	0.15	18.90	23.40	9.94
SE	17.7	0.07	0.03	–	0.8	–	0.07	0.03	0.03	4.12	4.87	2.66
Median	580	3.58	3.81	–	5.0	–	1.00	3.40	3.53	213	219	224
Range	405	1.58	1.23	–	16.0	–	2.00	0.70	0.65	67.0	69.0	32.0
Cohort 2 (1978–1991) (N = 65)												
Mean	43.2	3.65	3.65	–	6.0		0.70	3.51	3.60	221.2	215.0	212.5
SD	4.6	0.36	0.38	–	3.8		0.46	0.30	0.37	18.50	20.31	18.36
SE	0.6	0.05	0.05	–	0.5	_–_	0.06	0.04	0.05	2.92	3.09	3.02
Median	43	3.75	3.84	–	4.0		1.00	3.53	3.67	222.0	216.0	218.0
Range	23	1.72	1.46	–	15.0	–	1.00	1.34	1.40	84.0	79.0	80.0
Cohort 3 (1992–2003) (N = 56)												
Mean	33.9	3.71	3.81	0.33	7.6	3.90	0.77	3.59	3.60	226.2	224.2	218.5
SD	3.3	0.24	0.19	0.48	6.0	0.79	0.43	0.27	0.31	17.65	24.50	17.07
SE	0.4	0.03	0.03	0.10	0.8	0.09	0.06	0.04	0.04	2.79	3.87	3.17
Median	33	3.77	3.87	0.00	6.0	3.90	1.00	3.69	3.59	223.0	224.5	217.0
Range	17	1.10	0.95	1.00	32.0	2.80	1.00	1.00	1.25	73.0	92.0	70.0

uGPA = undergraduate grade point average, gsGPA = graduate school grade point average

Fellowship = predoctoral fellowship/other independent award

Pubs = publications resulting from MD–PhD training

Time-to-PhD defense = total time in years of graduate research training from beginning of full-time research to dissertation defense date

Career outcome = first full-time independent position following completion of MD–PhD training

mspGPA = medical school preclinical grade point average

mscGPA = medical school clinical grade point average

Step 1, 2, 3 = USMLE Step 1, 2, or 3 score

During the period encompassing this study, the first 2 years of the VUSM curriculum consisted almost exclusively of preclinical biological sciences coursework. We used the GPAs for medical school years one and two to calculate a mean preclinical GPA (mspGPA). The final 2 years of the medical curriculum consisted primarily of clinical clerkships and electives with little didactic coursework. Therefore, we used the GPAs for medical school years three and four to calculate a mean clinical GPA (mscGPA). USMLE Step 1, 2, and 3 scores were available for the majority of students.

The gsGPA represents the cumulative GPA for each student during the entire interval of graduate training (scale of 0–4). Cohort 1 MD–PhD students graduated from the Departments of Biochemistry, Cell Biology, Microbiology and Immunology, Molecular Physiology and Biophysics, Pathology, and Pharmacology. Cohort 2 students graduated from those departments as well as the Departments of Biomedical Engineering and Molecular Biology. Cohort 3 students graduated from cohort 1 and 2 departments as well as the Departments of Biological Sciences, Cancer Biology, and the Program in Neurosciences. About 30 % of cohort 1 uGPAs and gsGPAs were originally scaled from 0 to 3. These GPAs were adjusted such that all GPAs were placed on a four-point scale to allow comparison within cohorts.

We analyzed a subgroup of cohort 3 students (N = 37) for which we could determine whether the student received a predoctoral fellowship from either a government or private agency. The results were scored using a binary scale, with receipt of a fellowship or grant scored a “1” and non-receipt scored a “0.”

We searched www.PubMed.gov for student publications during the years of enrollment in the MSTP plus 5 years following graduation, as graduates were listed as publication authors for several years after completion of PhD training. We analyzed only the total number of publications and did not account for impact factor.

Information about time-to-dissertation defense was made available for a subset of cohort 3 (N = 48) using an electronic database maintained by the VUSM Office of Biomedical Research, Education, and Training. We used time-to-defense as a study metric rather than time to awarding the PhD degree since many students returned to medical school shortly after the thesis defense yet did not formally receive the PhD degree until several months later. The time-to-defense interval begins at the initiation of full-time graduate research, usually July 1 following the completion of the first 2 years of medical school and the USMLE Step I exam, and ends on the date of the dissertation defense. We converted the interval between the two dates into years.

Career outcome of MSTP graduates was defined as the first full-time position after completion of residency or fellowship training. Career outcome was scored using a binary scale, with an academic position with a primary appointment in a clinical or basic science department, a research position at a government institution such as the Centers for Disease Control, National Institutes of Health, or other government research organization such as the Department of Defense or Food and Drug Administration, or a research position in a biotech firm or the pharmaceutical industry scored a “1.” Any other position, including private practice, consulting, or non-academic healthcare, was scored a “0.”

### Statistical analysis

Multiple linear regression and logistic regression were used to assess associations between predictor variables (MCAT score and uGPA) and outcomes of interest (mspGPA, gsGPA, mscGPA, USMLE Step 1, 2, and 3 scores, publication number, and career outcome) for all cohorts. Additionally, we segmented the MCAT subsections of the Biological Science, Physical Science, and Verbal Reasoning scores and regressed outcomes against these variables for cohort 3. Cohort 3 also included predoctoral fellowships and time-to-PhD defense as additional outcomes. We did not combine the three cohorts and instead analyzed each group independently to account for the different scoring methods used for each version of the test as well as to mitigate changes over time with undergraduate, medical, and graduate GPAs. Statistical analyses were conducted using R version 3.0.2. on a Windows operating system.

## Results

The results of regression analyses to determine whether the MCAT predicts unique MD–PhD student training and career outcome measures are shown in Table 2, which include gsGPA, predoctoral fellowship receipt, publication number, time-to-defense (both outcomes for a subset of cohort 3 only), and type of first career position after completing training. Results of analyses of medical school performance (mspGPA, mscGPA, and USMLE Step I, II, and II scores) are shown in Table 3.

**Table 2:** Predictive validity of three versions of the Medical College Admission Test for 153 graduates of the Vanderbilt Medical Scientist Training Program

Cohort (Matriculation years) (N)	gsGPA			Fellowships^e^			Publications			Time to PhD defense			Career outcome		
	β^b^	CI	*p* value	β	CI	*p* value	β	CI	*p* value	β	CI	*p* value	β	CI	*p* value
Cohort 1 (1963–1977) (N = 32)															
uGPA	***0.33***	***[−0.63, 0.33]***	***0.04***		N/A		−2.97	[−7.86, −1.92]	0.24				0.43	[−2.88, −3.45]	0.78
MCAT^a^	0.0003	[−0.001, −0.002]	0.75				0.01	[−0.01, −0.04]	0.30				−0.002	[−0.02, −0.01]	0.79
Cohort 2 (1978–1991) (N = 65)															
uGPA	**0.25**	**[−0.51, −0.01]**	**0.06**		N/A		1.85	[−4.39, 0.68]	0.16		N/A		**−**0.86	[**−**2.74, **−**0.71]	0.32
MCAT^c^	−0.001	[**−**0.02, **−**0.02]	0.91				***−0.25***	***[−0.44, −0.05]***	***0.02***				0.01	[**−**0.11, **−**0.12]	0.93
Cohort 3 (1992–2003) (N = 56)															
uGPA	***0.23***	***[−0.43, 0.03]***	***0.03***	1.12	[−1.72, −4.43]	0.46	−2.25	[−8.85, −4.35]	0.51	−0.368	[−1.019, 0.283]	0.27	−0.41	[−3.94, −2.57]	0.80
MCAT^d^	−0.0002	[−0.01, 0.01]	0.98	0.001	[−0.19, 0.19]	0.99	0.07	[−0.39, −0.52]	0.77	0.035	[−0.018, 0.087]	0.20	−0.09	[−0.30, −0.11]	0.35
uGPA	**0.20**	**[**−**0.02,** −**0.42]**	**0.08**	−0.04	[−3.34, −3.46]	0.98	−4.41	[−11.61, −2.78]	0.24	−0.335	[−1.000, 0.330]	0.33	−1.53	[−5.84, −1.78]	0.42
MCAT (PS)^f^	−0.01	[−0.04, −0.02]	0.65	−0.27	[−0.75, −0.16]	0.24	0.09	[−0.88, −1.06]	0.84	0.060	[−0.048, 0.167]	0.28	−0.26	[−0.82, −0.20]	0.30
MCAT (BS)	0.01	[−0.03, −0.05]	0.67	−0.04	[−0.69, −0.59]	0.91	−0.48	[−1.73, −0.75]	0.45	0.040	[−0.095, 0.175]	0.56	−0.19	[−0.76, −0.36]	0.50
MCAT (VR)	0.02	[−0.01, −0.06]	0.22	0.22	[−9.38, −0.89]	0.48	0.47	[−0.71, −1.65]	0.44	−0.004	[−0.116, 0.109]	0.95	−0.03	[−0.56, −0.49]	0.91

^a^MCAT subscores between 1963 and 1977 consisted of Science Achievement, General Information, Quantitative Ability, and Verbal Ability

^b^Numbers indicate slope (β), confidence interval (CI) and *p* Value. Italicized numbers indicate statistically significant associations, and bold numbers indicate associations approaching significance

^c^MCAT subscores between 1978 and 1991 consisted of Biology, Chemistry, Physics, Science Problem Solving, Quantitative Skills, and Reading Skills

^d^MCAT subscores between 1992 and 2003 consisted of Biological Sciences, Physical Sciences, and Verbal Reasoning

^e^Population (N = 37) of Cohort 3 only beginning with first year of graduates who had cited an award of a predoctoral fellowship/grant

^f^MCAT *PS* Physical Sciences, *BS* Biological Sciences, and *VR* Verbal Reasoning subscores

**Table 3:** Predictive validity of three versions of the Medical College Admission Test for 153 graduates of the Vanderbilt Medical Scientist Training Program

Cohort (Matriculation years) (N)	mspGPA			mscGPA			Step I Score			Step II Score			Step III Score		
	β^b^	CI	*p* value	β	CI	*p* value	β	CI	*p* value	β	CI	*p* value	β	CI	*p* value
Cohort 1 (1963–1977) (N = 32)															
uGPA	***0.22***	***[0.04, −0.41]***	***0.03***	0.09	[−0.06, −0.25]	0.24	1.88	[−24.34, −28.10]	0.89	−4.21	[−31.90, −23.48]	0.77	−0.06	[−11.55, −11.44]	0.99
MCAT^a^	−**0.0011**	0.002, −0.000I	**0.08**	0.0001	[−0.001, 0.001]	0.89	−0.03	[−0.17, −0.10]	0.63	0.07	[−0.07, −0.22]	0.34	0.02	[−0.06, −0.10]	0.60
Cohort 2 (1978–1991) (N = 65)															
uGPA	***0.32***	***[0.12,*** *−* ***0.51]***	***0.003***	***0.34***	***[0.09,*** *−* ***0.59]***	***0.01***	−1.83	[−19.87, −16.23]	0.84	6.27	[−12.18, −24.72]	0.51	5.73	[−13.20, −24.67]	0.56
MCAT^c^	0.003	[−0.01, −0.02]	0.66	0.004	[−0.01, −0.02]	0.65	0.74	[−0.50, −1.97]	0.25	***1.36***	***[0.09,*** *−* ***2.63]***	***0.04***	1.01	[−0.18, −2.20]	0.11
Cohort 3 (1992–2003) (N = 56)															
uGPA	0.25	[−0.07, −0.57]	0.13	0.09	[−0.26, −0.44]	0.61	11.34	[−12.09, −34.77]	0.35	**28.95**	**[**−**1.22,** −**59.12]**	**0.07**	9.40	[−20.78, −39.59]	0.55
MCAT^d^	−0.001	[−0.02, −0.02]	0.94	***0.03***	***[0.01,*** *−* ***0.06]***	***0.02***	0.48	[−1.25, −2.20]	0.59	1.43	[−0.92, −3.77]	0.24	0.27	[−1.62, −2.17]	0.78
uGPA	0.15	[−0.20, −0.45]	0.41	0.09	[−0.32, −0.51]	0.66	12.39	[−11.75, −36.53]	0.32	**33.06**	**[**−**0.35,** −**66.47]**	**0.06**	2.56	[−29.76,−34.88]	0.88
MCAT (PS)^e^	0.0001	[−0.05, −0.05]	0.99	0.01	[−0.05, −0.07]	0.74	−0.41	[−3.88, −3.07]	0.82	−2.95	[−7.78, −1.89]	0.24	−**3.23**	**[**−**6.659,** −**0.195]**	**0.08**
MCAT (BS)	−0.04	[−0.10, −0.03]	0.25	−0.01	[−0.09, −0.07]	0.82	−0.45	[−4.60, −3.71]	0.84	3.69	[−2.37, −9.76]	0.24	1.98	[−2.578, −6.534]	0.40
MCAT (VR)	0.03	[−0.03, −0.09]	0.34	0.03	[−0.04, −0.09]	0.46	2.34	[−2.20, −6.89]	0.32	0.66	[−5.63, −6.94]	0.84	3.14	[−2.268, −8.549]	0.27

^a^MCAT subscores between 1963 and 1977 consisted of Science Achievement, General Information, Quantitative Ability, and Verbal Ability

^b^Numbers indicate slope (p), confidence interval (CI) and *p* value. Italicized numbers indicate statistically significant associations, and bold numbers indicate associations approaching significance

^c^MCAT subscores between 1978 and 1991 consisted of Biology, Chemistry, Physics, Science Problem Solving, Quantitative Skills, and Reading Skills

^d^MCAT subscores between 1992 and 2003 consisted of Biological Sciences, Physical Sciences, and Verbal Reasoning

^e^MCAT *PS* Physical Sciences, *BS* Biological Sciences, and *VR* Verbal Reasoning subscores

### Graduate school GPA

An association between MCAT score and gsGPA approaching statistical significance was found in cohort 2 (β = 0.25, *p* = 0.06) and in cohort 3 (β = 0.20, *p* = 0.08). There was a significant association between the uGPA and the gsGPA for cohorts 1 and 3 (β = 0.33, *p* = 0.04 and β = 0.23, *p* = 0.03, respectively).

### Predoctoral fellowship

There was no significant association of either MCAT score or uGPA with cohort 3 predoctoral fellowships with *p* values ranging from 0.24 to 0.99.

### Publications

There was a significant association of MCAT score and number of publications for cohort 2, but the correlation was negative (β = −0.25, *p* = 0.02). There was no significant association of uGPA and number of publications for any cohort.

### Time-to-PhD defense

There was no significant association of MCAT or uGPA with time-to-defense for any cohort with *p* values ranging from 0.20 to 0.95.

### Career outcome

There was no significant association of either MCAT score or uGPA with career outcome for any cohort with *p* values ranging from 0.30 to 0.91.

In examining MD–PhD student medical school performance, we found significant associations of the MCAT and uGPA with several outcome measures (Table 3).

### Preclinical GPA

There was no significant association of MCAT and mspGPA, although a weak negative association approached significance for cohort 1 (β = −0.0001, *p* = 0.08). There was a significant association of uGPA and mspGPA for cohorts 1 and 2 (β = 0.22, *p* = 0.03 and β = 0.32, *p* = 0.003, respectively).

### Clinical GPA

There was a significant association of MCAT and mscGPA for cohort 3 (β = 0.03, *p* = 0.02,) and uGPA and mscGPA for cohort 2 (β = 0.34, *p* = 0.01).

### USMLE scores

There was no significant association of MCAT score or uGPA for performance on the Step I exam for any cohort with *p* values ranging from 0.25 to 0.89. There was a significant association of MCAT score and Step II score for cohort 2 (β = 1.36, *p* = 0.04). An association of uGPA and Step II score for cohort 3 approached significance (β = 28.95, *p* = 0.07). There was no significant association of MCAT score or uGPA for performance on the Step III exam for any cohort.

Combination of uGPA and MCAT score did not yield signification associations with any medical school outcome with the exception of the USMLE Step II and Step III scores for cohort 3.

## Discussion

In this study, we analyzed the validity of the MCAT in predicting training performance measures and career outcomes for 153 MD–PhD students who matriculated into the Vanderbilt MSTP between 1963 and 2003. The students were divided into three cohorts corresponding to the different versions of the MCAT used during the observation interval. Our findings suggest that the MCAT does not predict MD–PhD outcomes.

The MCAT version used for cohort 1 students (matriculating between 1963 and 1977) was designed “to encourage a broad-based liberal education” for applicants (McGaghie 2002). Predicting success in the basic medical sciences was stated to be a “limited objective” of the test. Consequently, the validity of that version of the MCAT for predicting physician-scientist trainee outcomes would be expected to be lower than subsequent versions. Additionally, cohort 1 and portions of cohort 2 applicants to the Vanderbilt MSTP were screened for admission with greater emphasis placed on “prior research experience and letters of recommendation” (Vanderbilt Medical Scientist Training Program 1987; Vanderbilt Medical Scientist Training Program 1992). For that cohort of students, the uGPA was a slightly more consistent predictor of MD–PhD performance in graduate school and the medical school preclinical curriculum than the MCAT.

The MCAT version used for cohort 2 students (matriculating between 1978 and 1991) incorporated changes to improve prediction of future clinical abilities and de-emphasized knowledge of non-science subjects (McGaghie 2002). However, our analysis showed that uGPA was a better predictor than the MCAT of academic performance. For this cohort of trainees, gsGPA, mspGPA, and mscGPA were significantly associated with uGPA, but none of these parameters was associated with MCAT score. However, MCAT was a more reliable predictor of USMLE Step II performance. Interestingly, MCAT was associated with number of publications for this cohort, but a higher MCAT score was associated with fewer publications.

The MCAT version used for cohort 3 students (matriculating between 1992 and 2003) further emphasized biological and physical sciences (Vanderbilt Medical Scientist Training Program 1992). The Verbal Reasoning section of the exam was intended to assess comprehension, application, and analysis of passages on humanities, social sciences, and natural sciences (McGaghie 2002). The most significant change to this version of the test was the inclusion of a writing subtest, subsequently shown to have only modest predictive validity for medical students with the possible exception of predicting clinical performance (Gilbert et al. 2002; Kreiter and Kreiter 2007). This version of the MCAT did not correlate more closely with MD–PhD training metrics than previous versions of the test, except for mscGPA, which showed a significant association. Pairing MCAT score and uGPA did not increase the association with most outcome measures, unlike previous studies (Julian 2005; Donnon et al. 2007; Callahan et al. 2010). Only USMLE Step II and Step III scores were significantly associated with the combination of MCAT score and uGPA.

We considered the possibility that MCAT averages of MD–PhD students might be significantly higher than those of MD students, which might account for the finding that MCAT scores are a reasonable predictor of MD student outcomes in comparison to our findings for MD–PhD students at Vanderbilt. During the study interval, MCAT scores were available for Vanderbilt MD students only for cohort 3 (1992–2003). For this cohort, mean MCAT scores were 32.93 for MD students and 33.98 for MD–PhD students, which do not differ significantly. Therefore, MD students and MD–PhD students appear to vary in a meaningful way that is uncovered by differences in the predictive validity of the MCAT. We also thought that the lack of predictive validity of MCAT score and uGPA might be explained by underfitting the logistic and linear models. Consequently, we explored a variety of alternative statistical approaches, including regressions with complex interaction terms and restricted cubic splines as well as random forests models. Our attempts to find significant associations did not differ substantially from the linear and logistic models.

In November 2011, the 5th Comprehensive Review of the MCAT (MR5) released recommendations for the first changes to the exam in more than 20 years. The revised examination will retain two natural science sections and include new sections to assess the psychological, social, and biological foundations of behavior and critical analysis and reasoning skills. The goal of the revision is to “prompt students to read broadly as they prepare for medical school.” The writing section will be eliminated. This new version of the MCAT is now available for MD and MD–PhD program applicants (Association of American Medical Colleges n.d.b.). Perhaps the revised MCAT will be a more effective predictor of MD–PhD student outcomes.

### Study limitations

There are important limitations to our study. The analysis was conducted using data from a single institution and may not be applicable to other MD–PhD programs. However, the metrics employed here should be useful to other programs for examination of their student populations and thus guide future research using larger datasets. Students analyzed in our study matriculated over a 40-year interval in which several changes were made in admissions procedures and the MD–PhD training curriculum. For example, there were three versions of the MCAT used, uGPA and gsGPA were calculated using different scales, and admissions committee membership changed over time. Because of these variables, we analyzed each cohort independently. Only scores earned by program matriculants were analyzed. Therefore, our study cannot be generalized to the predictive validity of MCAT scores outside the range of those used in our sample.

### Topics for future research

Our study suggests that within the scores earned by MD–PhD program matriculants at Vanderbilt, the MCAT is not an effective predictor of important training outcomes for MD–PhD students, including gsGPA, number of publications, time-to-PhD defense, and first independent position following training. Therefore, the MCAT appears to be an insensitive metric for assessing an applicant for a career as a physician-scientist. Moreover, our data suggest that the MCAT is not an optimum metric for evaluating the quality of a matriculating class of MD–PhD students or the overall strength of an MD–PhD program. MCAT scores and uGPAs contribute to the rankings of medical schools by various organizations and periodicals like the *U.S. News and World Report* (Flanigan and Morse 2014). However, since these parameters are not predictive of key training outcomes, alternative metrics should be sought for these types of rankings.

What might better predict MD–PhD training metrics and career outcomes than MCAT and uGPA? We suggest that several prematriculation variables may have a synergistic effect on MD–PhD student performance measures and career choice. For example, we think that scientific publications prior to MD–PhD program entry, length and depth of prematriculation research experiences, grants and fellowships received prior to matriculation, the quality of letters of recommendation, and undergraduate science awards more accurately predict MD–PhD student training performance. Therefore, we have initiated a detailed follow-up study to evaluate whether these and other prematriculation and postmatriculation variables are more effective predictors of training metrics and career outcomes.

Other prematriculation variables that may correlate with MD–PhD training outcomes are leadership, teamwork, and perseverance (Duckworth et al. 2007; McGee and Keller 2007; Tough 2011). Therefore, beginning with the entering class of 2007, we have sought to assess these qualities in applicants to the Vanderbilt MSTP by requesting an essay describing a significant challenge overcome by the applicant. Responses to this question are considered when screening applications, preparing for interviews, and evaluating candidates for admission. We do not have sufficient data to permit definitive conclusions about the predictive validity of this essay. However, of the 80 students who have matriculated into the Vanderbilt MSTP since 2007, only four have left the program prior to receiving MD and PhD degrees, half the national attrition rate for MD–PhD programs cited in Brass et al. (2010). Moreover, we think it likely that the curricular elements developed for physician-scientist training at this institution contribute to retention, positive training metrics, and successful career outcomes. For example, the Vanderbilt MSTP has an extensive network of student-advising functions that span the duration of medical and graduate training and the transitions between these curricular phases. We also incorporate formal training in scientific inquiry, leadership, and career development, among other topics, into the MD–PhD curriculum. It may take several more years of study before we can identify the factors that are most significant.

The substantial investment of extramural and institutional funds to support MD–PhD programs has led to the training of a large cohort of productive physician scientists (Andriole et al. 2008; Brass et al. 2010). It is essential that those in leadership positions determine the factors that best predict success of program applicants. Identification of such factors will allow individualized professional training and personal development for dual-degree students. This approach should result in substantial contributions to the structure of the national physician-scientist workforce.
